# Covert use of contraception in three sub-Saharan African countries: a qualitative exploration of motivations and challenges

**DOI:** 10.1186/s12889-020-08977-y

**Published:** 2020-06-05

**Authors:** Simon P. S. Kibira, Celia Karp, Shannon N. Wood, Selamawit Desta, Hadiza Galadanci, Fredrick E. Makumbi, Elizabeth Omoluabi, Solomon Shiferaw, Assefa Seme, Amy Tsui, Caroline Moreau

**Affiliations:** 1 Department of Community Health and Behavioral Sciences, School of Public Health, Makerere University, Kampala, Uganda; 2 Department of Population, Family and Reproductive Health, Johns Hopkins Bloomberg School of Public Health, Baltimore, USA; 3 Center for Advanced Medical Research and Training, Bayero University Kano, Kano State, Nigeria; 4 Department of Epidemiology and Biostatistics, School of Public Health, Makerere University, Kampala, Uganda; 5 Centre for Research Evaluation Resources and Development, Ile-Ife Osun State, Nigeria; 6 Department of Reproductive Health and Health Service Management, School of Public Health, Addis Ababa University, Addis Ababa, Ethiopia; 7 Soins et Santé Primaire, CESP Centre for Research in Epidemiology and Population Health, U1018, Inserm, F-94807 Le Kremlin-Bicêtre, France

**Keywords:** Contraception, Covert use, Partner dynamics, Decision-making, Empowerment

## Abstract

**Background:**

The balance between increasing men’s participation in family planning and rights-based initiatives favoring women’s empowerment is highlighted with the issue of covert use of contraception. While covert use has been documented in low- and middle-income countries as a way for women to obtain contraception in light of partner opposition, little is known about women’s decision-making processes, actions, and potential consequences of discreet contraceptive use. We aimed to understand women’s choices to use contraception covertly and the challenges they faced in concealing their use across three sub-Saharan African countries.

**Methods:**

Women aged 15–49 and their male partners were purposively sampled from urban and rural sites in Ethiopia, Northern and Southern Nigeria, and Uganda for 120 in-depth interviews and 38 focus group discussions. Semi-structured interviews explored women’s and girls’ empowerment surrounding sex, childbearing, and contraception. Interviews were conducted in local languages, audio-recorded, and transcribed verbatim into English. Inductive thematic analysis was used to analyze data; covert use codes were reviewed and matrices were created based on themes and sub-themes.

**Results:**

Findings comprised three thematic areas: the practice of covert contraceptive use and reasons for using covertly; challenges for women who use contraception covertly; and consequences of disclosure or being discovered. While some women initiated using contraception covertly due to tensions within relationships or to keep peace within the home due to known partner opposition, others did not consider family planning to be a male responsibility. Though covert use was commonly discussed, it was also socially sanctioned, and portrayed as an act of female disobedience that questioned the social order of patriarchy. Further challenges of using covertly included lack of financial and social support, and suspicions surrounding delayed fertility and contraceptive-related side effects. Repercussions comprised increased suspicion, threats, or violence, though some women reported improved couple communication with disclosure.

**Conclusions:**

Results indicate that while covert use of contraception is common, continued covert use is challenging, especially when side effects manifest. Covert use may further suggest women taking independent action, symbolizing some level of empowerment. Results underscore the importance of disentangling unique reasons for covert use and the severity of repercussions of disclosure.

## Background

The slow pace of fertility transitions in sub-Saharan Africa has re-energized international interest in family planning as a prioritized strategy to improve maternal health. In particular, a series of commitments launched with the 2011 Ouagadougou Partnership, followed by the 2012 London Summit, agreed to take concerted action and make contraception available to an additional 120 million women in low- and middle-income countries (LMICs) by 2020 [1, 2]. Most programmatic efforts to improve contraceptive use have focused on women, as they bear the greatest burden of pregnancy-related health issues and are the primary users of modern contraceptive methods [3–5]. However, recognizing that gender norms inform sexual and reproductive health (SRH) decisions [6–9], there is growing interest in engaging men in the SRH agenda [9–11]. Couple-based reproductive health programs seem a promising approach to improving acceptability and use of contraception in patriarchal societies; yet, they may also undermine women’s rights to control their own bodies, including women's free choice to use or not use contraception [12]. The delicate balance between pragmatic couple-based approaches and a rights-based perspective favoring women’s empowerment is brought to the fore with the issue of covert use of contraception (i.e. the practice of using contraception without one's partner’s knowledge [12–14]).

Most of the current literature on covert use of contraception falls in two categories: qualitative studies of women’s reasons for and experiences of using contraception without their partner’s knowledge [9, 15–18] or quantitative assessments of covert use from Demographic and Health Surveys (DHS) inferred from discordant reports of modern method use reported by the wife, but not the husband [19, 20]. Two mixed-methods studies have begun to bridge the gap between qualitative and quantitative research by investigating levels of and reasons for discreet contraceptive use in facility-based or cohort populations [13, 14].

Covert use of contraception reveals the tensions emerging from the dissociation between sexuality and procreation, and the repercussions of this division on gender power dynamics. Qualitative studies have documented women’s reasons for concealing or wanting to conceal their use of contraception, including their discomfort or lack of confidence in initiating discussions of sexual issues, male opposition to contraception, and strong pronatalist culture that values high fertility [8, 9, 15, 21–23]. Further, studies exploring women’s acceptability of new contraceptive methods, such as patches and injectables, have highlighted women’s preferences for discreet forms of contraception [24–26]. At the couple level, a lack of economic support for childrearing or perceived neglect in the marriage often trigger such decisions, as women seek to gain more independence in the context of family instability [9, 18].

At a broader level, covert use of contraception represents a strategy for women to challenge opposition to family planning, without suffering the related social consequences [9, 18, 21]. However, women are often faced with the challenge of balancing the physical and social benefits of using contraception covertly with the costs of concealing their use and the potential consequences of their discovery. Qualitative studies in West and East Africa suggest higher discontinuation rates among “covert” as opposed to “overt” users [18, 27–29]; however, there is a lack of longitudinal evidence to examine the actual effect of covert use on contraceptive dynamics, including discontinuation. In addition, studies have demonstrated that women fear partner-perpetrated violence, threats of marriage dissolution, neglect, and social sanctions in the event that their partners learn of their contraceptive use [13, 18, 23, 28]. While several studies have examined women’s reasons for using covertly [12–14, 16, 18, 23, 30], a dearth of literature remains surrounding the comprehensive decision-making processes that women engage in to initiate and continue using contraception covertly. Further, the ways in which women balance the benefits of contraceptive use and the potential consequences of their use being discovered, and how these decisions relate to empowerment or disempowerment, are not well explored in the literature.

The current study builds on a larger cross-cultural project focused on measuring women’s and girls’ empowerment (WGE) in SRH in sub-Saharan Africa. In order to fill the gaps in the knowledge base surrounding covert use decision-making processes, including trade-offs of benefits and potential consequences, we utilized women’s and men’s perspectives and experiences to evaluate how covert use practices varied according to cultural and residential contexts in three sub-Saharan African countries. The specific objectives of these analyses were as follows: 1) to explore couple dynamics informing women’s decisions to conceal their use of contraception; 2) to understand the challenges that women face when using contraception covertly; and 3) to explore women’s fears related to disclosing covert use of contraception and/or being discovered.

## Methods

The Women’s and Girls’ Empowerment in Sexual and Reproductive Health (WGE-SRH) qualitative study was conducted in three countries across four equally-represented sites. The four study sites, included 1) the Amhara region of Ethiopia, 2) Anambra state in Southern Nigeria, 3) Kano state of Northern Nigeria, and 4) Mukono and Iganga districts of Uganda. Sites were identified based on long-standing research collaborations on the PMA2020 project, now called PMA (www.pmadata.org), between Johns Hopkins Bloomberg School of Public Health and partner universities/research institutions. Further, these sites represent a range of East and West African cultures at different stages of fertility transitions. Specifically, Nigeria and Uganda exhibit high total fertility rates with slow declines (TFR; 5.3 and 5.4, respectively), while Ethiopia has shown steady fertility decline with TFR dropping from 5.4 in 2005 to 4.2 in 2016 [31–33]. The Anambra state in Southern Nigeria and the Kano state in Northern Nigeria served as distinct sites for this study given substantial geographical and SRH indicators, including age at marriage, polygyny, fertility, and family planning uptake differences.

A total of 38 focus group discussions (FGDs) and 120 in-depth interviews (IDIs) were conducted from July–August 2017 among women aged 15–49 years and men aged 18 and older, selected from urban and rural communities in each site. A total of 440 participants (120 via in-depth interviews and 320 in FGDs) were interviewed. Participants were purposively sampled according to age, marital status and area of residence within each site. A common cross-country research protocol was developed and implemented following qualitative research training in each site. The study was carried out in collaboration with in-country researchers from the PMA2020 project [34], who managed all recruitment, data collection, transcription, translation, and coding activities. Institutional Review Board (IRB) approval was obtained at both Johns Hopkins Bloomberg School of Public Health (JHSPH), USA and in each country: Addis Ababa University in Ethiopia, Anambra Ministry of Health and Bayero University Kano in Nigeria, and Makerere University School of Public Health in Uganda. IRB guidelines were followed in each. Consent to participate was written and assent oral in Uganda and Nigeria per Makerere University, Anambra Ministry of Health, and Bayero University Kano IRB guidelines; consent was oral and assent written in Ethiopia per Addis Ababa University IRB guidelines. In-depth qualitative study procedures are further described elsewhere [16, 35].

In-country teams used purposive sampling (by age, marital status, and area of residence) within each site to recruit women and men for both FGDs and IDIs. Eligibility criteria included women aged 15–49 (or men whose wives were 15–49 years) who resided within the study area. Eight female FGDs and two male FGDs were conducted in each of four sites, except Anambra (where a total of eight FGDs were conducted). IDI participants comprised 12 couples, who were interviewed separately (12 females and 12 males) and six unmarried women, who were equally divided between urban and rural settings.

Semi-structured interviews were conducted to explore women’s sexual and reproductive autonomy, self-efficacy, decision-making, and negotiation strategies. All participants also completed a brief questionnaire regarding their background characteristics; responses allowed for the disaggregation of themes by demographic characteristics. Interviews were conducted by trained interviewers in local languages, audio-recorded, transcribed and translated verbatim, and uploaded in Atlas.ti. An independent quality control was conducted, where 15% of the audios files were transcribed again by an independent person in each site to check for consistency in the translations and completeness.

An inductive thematic approach was used for analysis. Investigators from each site read transcripts and identified emerging themes, which supported the generation of an initial codebook. The initial codebook was shared across sites and revised using an iterative process. Investigators in each site applied the core set of cross-country codes and developed site-specific sub-codes, as necessary. For the secondary analysis presented in this paper, codes related to covert use were extracted and matrixes used to examine patterns of covert use and responses to covert use across sites and among specific sub-groups (i.e. by sex and marital status).

## Results

The findings are presented under three thematic areas: the practice of covert contraceptive use and reasons for using covertly; challenges for women who use contraception covertly; and consequences of disclosure or being discovered.

### The practice of covert use and reasons for using covertly

Covert use of contraception was evoked by men and women across sites, suggesting that clandestine use of family planning methods was a common practice.

Women that make the decision on their own to engage in family planning, even without the knowledge of their husband [women who use covertly], are more compared to those that seek their husband’s consent. Men do not know the hardship women face, but they just see them with children.

*-- Female FGD participant, Married, Urban Kano (Nigeria)*

Covert use was facilitated by the diffusion of concealable female-controlled methods, such as the injectable and implant, although other methods, such as oral contraceptive pills or emergency contraception, were also mentioned, particularly for young women whose partners opposed family planning: “Once the man really wants to fail you, you can stealthily take pills or go for an injection since it is not a scar, which can be easily noticed” (*Female FGD participant, Married, Rural Uganda*).

Unmarried women or women who were not living with a partner, also described the potential of using more traditional methods, such as fertility awareness/rhythm covertly. However, the concealment of these traditional methods was challenging in unions, given many women's feare of discovery.

The woman can stealthily get themselves enrolled on family planning without the notice of the husband. In addition, she can also be calculative and know her safe days where she could try to avoid getting pregnant [fertility awareness method].

*--Female FGD participant, Married, Rural Uganda*

Both women and men indicated a number of influential factors leading to the covert use of contraception. In particular, couple dysfunction, including gender power imbalance and volatile environments in the relationships, such as alcohol abuse and intimate partner violence, prevented women from asserting their reproductive preferences. Women and men provided examples of relationship instability propelling covert use, including suspicions of infidelity and absence of men’s financial support to the family. In such cases, covert use could be a strategy to distance oneself from a failing relationship by preventing another birth that would increase partner dependency.

Some men take alcohol, so [they] cannot make decisions. He is always drunk, and you cannot sit and talk. He sleeps with you when he is drunk. You cannot talk to him about family planning. Some men are always busy. They do not have time to sit and talk. Whenever you want to talk, he does not have time to talk. So, you just go for family planning. If he asks me why don’t you give birth? I say ‘I do not know, may be my fallopian tube has problems’.

*-- Female FGD participant, Married, Urban Uganda*

Beyond overt conflict, many women avoided direct communication about family planning because they sought to evade husband opposition and keep “harmony” in the relationship: “I will not say ‘no’ to him to avoid his sadness and quarrel. But, I will use the family planning method without him. I will delay like that” *(Female IDI participant, Married, Urban Ethiopia).*

Further, several men were aware about the practice in their communities and a few even suspected their own partners to be using covertly.

Most women in this community use family planning secretly. The man knows it after some time. Like my wife used it without my knowledge, but when she told me about it, I just told her ‘it is ok’…most women decide for themselves what family planning to use.

*-- Male FGD participant, Urban Uganda*

In fact, some men recognized covert use as women’s exercise of power to overcome male dominance over reproductive decisions.

I think it is the wife, because it is the women who decide to produce for a man any number of children wanted. Even if a man wants and a woman does not want, she will not get pregnant since they have many methods to use. I used to force one of my wives to produce more children not knowing she had an implant. It took me four months to know that she had an implant, I was touching her arm and felt it. When I asked her about it, she told me she had already gone for family planning since the child was still young and it [the implant] was to last three years. Then I told her it was okay although she had not told me. So, a wife can start taking even pills without your knowledge. So, it is the woman who decides to produce for a man when she wants.

*-- Male FGD participant, Urban Uganda*

While frequently reflective of a lack of support in women’s reproductive decision-making, covert use could be an expression of women’s ultimate autonomy in contraceptive decisions. Indeed, some women and men assumed that women asserted full responsibility for family planning decisions and considered their partners had no contributions to these matters.

I believe it is the wife who has the final say. She is the one who decides on the number of children to produce since she is the one who goes to labor ward. She can say I felt a lot of pain for this child, let me spend three years without conceiving, she can decide to swallow pills when the husband does not know.

*--Male FGD participant, Urban Uganda*

Further reflective of women’s autonomy, especially among young women at the start of a relationship, covert use was a strategy to test a new partner before committing to marriage.

Some women or girls now monitor the kind of man and family to get married to in the first place. Some now secretly use contraceptives before they even get married. After they get married, they can ensure that they can be taken care of in terms of feeding and training of their children, before they decide to start giving birth. That is what majority of women are afraid of now.

*-- Female FGD participant, Urban Kano, Nigeria*

### Challenges in women’s experience of using contraception covertly

While commonly discussed, covert use was also socially sanctioned, as it challenged existing gender power structures by defying male authority. More than conflicting pronatalist social norms, still prevalent in many settings, covert use was often portrayed as an act of female disobedience, questioning the social order of patriarchy. This was shared in a focus group discussion among men in rural Ethiopia, who reflected on community shame over women who were discovered disobeying their husbands.

According to me, I don’t think it [covert use] is common because if her family later finds out, it’s a big insult. She also fears something may come from her husband and from elders and because she doesn’t want her family to hear about her shame and how she cheated and tricked her husband. In this context and this country’s standard, I don’t think this is common.

*-- Male FGD participant, Rural Ethiopia*

Besides challenging gender norms, women often lacked economic and social support to adhere to their contraceptive regimen. Particularly, women across sites mostly relied on financial support of partners to obtain methods and attend additional medical visits in case of contraceptive side effects.

You can secretly go for an injection without his knowledge. We used to do that way back, but got some problems that worried us a lot. So, there is the issue of secretly doing it—you get problems. So, you can imagine what can follow, yet for those problems to go, you have to be with money. So, where can you get the money when you have no job?

*-- Female FGD participant, Married, Rural Uganda*

A few women also described confidentiality as a limiting factor to access family planning services secretly, especially in small communities with strong social ties. Even if information was concealed from the husband, it was not necessarily hidden from other community members. Some women shared their experiences with confidantes, increasing their risk of disclosure.

For some women, when they discuss with their partners and they refuse, [the women] go for an injection or implant. And, when the husband asks, ‘Why are we not having our next child?’ she says ‘I do not know why. I think it is not yet time.’ A certain woman I know experienced this. That is what she told the husband, that ‘Let’s wait it seems it is not yet time. I may conceive in a short period of time.’ But she had started using family planning without the man’s consent. But someone informed the husband that your wife started using family planning, and the situation was not good.

*-- Female FGD participant, Married, Urban Uganda*

Men and women alike mentioned that it was possible for the male partner to obtain information about his spouse’s use of contraceptives from sources close to the woman, or that this information could be unintentionally disclosed if partners eavesdropped on conversations.

It happened sometime when I was sharing with a friend who came to my house [to visit], after she got married and had her first child. I shared with her what I was told to do after a woman’s first child. How if she does not take care she will not know when she will take in for the second one immediately. That the best thing is prevention, they say, is better than cure, and that she should better go and get the pill and protect herself. I brought out the pack of the pills and gave it to her. That was how my husband knew about my use of it. He shouted, ‘So, you have been using this in this house without my knowledge!’… Otherwise how could he explain that his wife had been taking such tablets right under his nose without his knowledge? Well, that was how he found out that I ever used that [pill].

*-- Female IDI participant, Married, Rural Anambra, Nigeria*

Women also discussed concerns regarding medical documentation, which could reveal the consultations they had in order to receive contraception. In addition, although rarely mentioned, some providers requested husband’s consent to use family planning before providing women with methods, which prevented some women from acting on their contraceptive preferences covertly.

It hurts them [men] because you may use contraceptives without the husband’s knowledge. Like me, my husband was [away] attending a training, so I went and started using contraceptives because I had a breastfeeding baby. When he returned, he told me, ‘Let us go to the village,’ and when we got there his parents told him, ‘It seems that girl is using contraceptives. Why isn’t she giving you a baby?’ then he said, ‘I haven’t been around, but she will give me a baby.’ Then I made a mistake of putting the family planning card in the bag, and as he was looking for something he read it, he quarreled, and told me, ‘It is time for you to pack up your things and go back to your [parents] home. How can you start using contraceptives in my absence and you didn’t even consult me?’ So, this caused quarrels, we had disagreements and his parents started mistreating me. When I felt that it was too much for me, I called home.

*-- Female FGD participant, Married, Urban Uganda*

A common concern related to covert use of contraception was that delayed pregnancy raised suspicion from husbands, families, and the larger community, as many women described that these parties wondered why women were not having children. Although seeking to delay or limit pregnancy was the reason women were using contraception covertly, it was not easy to sustain ‘excuses’ of not getting pregnant, while in an active sexual relationship. A few men also noted this as a means of suspecting their partners were using contraception covertly.

In my own understanding, a woman does not tell the husband that she wants to stop childbearing. Like what I have said before, it is the two of them [the couple] that will decide how you will end childbearing within yourselves because like I am now, if I need a child my wife cannot stop me. If I meet her [have sex] when I know she can conceive, and for three years nothing happens, you know that there will be problem in the family. I will question her, ‘Did you abort? Are you taking any drug? Did you do family planning?’

*-- Male FGD participant, Rural Anambra, Nigeria*

Women also highlighted the issue of physical side effects, including heavy bleeding, weight loss or gain, and lack of libido, which potentially unmasked their contraceptive use to their male partners. When the side effects were unbearable and likely to be noticed by their partners, some women were compelled to disclose their contraceptive use.

R1: Again I [can] secretly go for maybe a coil or pills, but the challenge is getting problems there. I remember one time I secretly went for an injection and bled heavily for four months. This made me decide to tell him, though he quarreled, the issues were solved. He did not kill me, but [disclosing] helped me to survive.

R2: Even me, I once secretly went, but as I speak now am not okay.

*-- Female FGD participants, Married, Rural Uganda*

Some men in Uganda reiterated the similar challenge of side effects leading to the unmasking of the women’s hidden use of contraception.

Family planning has got advantages and disadvantages. Men learn of it through the disadvantages like maybe not giving birth, yet a woman used to give birth; a woman who used to have normal periods and they start to become irregular; or if she is using pills, a man will see them and it usually brings violence.

*-- Male IDI participant, Rural Uganda*

### Consequences of disclosing or discovering covert use

Several possible scenarios were described by women and men upon disclosure or discovery of covert use. Women primarily discussed increased suspicions and mistrust that could compromise relationship quality and lead to potential couple dissolution.

If the wife uses family planning without telling him, number one, [she will lose] trust. His trust on her will decrease very much from previous. Their love in the house will also decrease. There is no problem if she used it by discussing with him, since they have done it with a mutual decision. But there will be quarrel because she does this without telling him and his trust on her will decrease.

*--Female FGD participant, Married, Urban Ethiopia*

Female participants also described fearing or experiencing violent reactions from partners, who could threaten or even beat them: “Some men hit them by saying, ‘Why do you hide from me?’ Conflict might happen because he could fight her morning and evening” (*Female IDI participant, Unmarried, Rural Ethiopia*). These reactions were exacerbated when women’s covert contraceptive practice was thought to conceal infidelity, thereby equating family planning methods use with extra-marital sexual relations.

‘How could you hide this from me?’ Eh, that means you are taking it because you have another thing [extramarital affair] or you are using it with another man. By saying those things, a disagreement could also occur. So, doing it by hiding is harmful for marriage. That is what I believe.

*-- Female FGD participant, Married, Urban Ethiopia*

In some cases, however, male participants suggested that disclosure could improve couple communication leading to agreement on family planning practices, and “permitting” women who were using contraception covertly to continue using it overtly. Some women also testified that, contrary to expectations, their partner did not negatively react when they learned they were using contraception covertly. “I did not tell him, but when he found out, he did not have any issue about it” *(Female IDI participant, Married, Rural Kano, Nigeria).* Such situations were described when men’s fertility intentions were aligned with women’s intentions, but the couple had failed to discuss family planning, thereby leading to covert use.

The man may be happy that his wife did not get pregnant because there are instances when men prefer women that are getting pregnant less frequently. It is true because she will give him more time, unlike the ones that are frequently getting pregnant. Physically, she will look more beautiful than a woman that is giving birth frequently. So, even though he did not accept her decision at first, he will like her more because of the time she is giving him and for her improved looks over the other women.

*--Female FGD participant, Married, Rural Kano, Nigeria*

## Discussion

This study explored the dynamics of women’s decisions to conceal their contraceptive use, the challenges they faced using contraception covertly, and the consequences of being discovered by partners relying on qualitative data gathered across three countries in sub-Saharan Africa. While women circumvented significant barriers to use contraception, lack of resources and social support, particularly from family and friends who could breach confidentiality, remained hurdles to women's continued use of methods, especially when women experienced side effects. Women’s general fear of consequences, related to their partners’ discovery of their use, constrained their autonomy over reproductive decisions.

Covert use of contraception was a common practice across all sites and residential settings for varied reasons, similar to some highlighted in other studies [13, 14]. However, despite the ubiquitous nature of this practice, it was sanctioned. Given the gender power dynamics in patriarchal settings [36–39] and the pronatalist norms and policies [12, 40] that are likely to prioritize women as reproducers, these sanctions are expected. As recognized across other contexts, gender power dynamics play a key role in limiting candid communication about SRH issues, including childbearing [8, 41]. This lack of communication, and sometimes uncertainty about a partner’s opinion on childbearing and pregnancy, may lead women to pursue more complicated or risky routes for achieving their reproductive aspirations. These narratives underscore the need for thorough contraceptive counseling for both individual women and couples, to ensure that contraceptive methods used both covertly and overtly maximize women’s priorities and take into account their unique and individual circumstances. Contraceptive counselling in the three study countries is still limited. For example, 75% of current users in Anambra, 40% in Kano, 56% in Ethiopia and 50% in Uganda reported not being told about potential side effects at the health facilities [42–44]. If covert contraceptive users are not well counseled when they obtain methods, these women are posed with more challenges on how to navigate contraceptive use successfully.

Although historically assumed to reflect a lack of autonomy requiring targeted intervention [14, 45, 46], our results indicate that covert use represents a spectrum of situations, ranging from coercion to full autonomy in contraceptive decisions. In all sites, some respondents considered contraception to be a woman’s prerogative and, accordingly, some female participants considered their partners' involvement non-essential in decision-making about contraception. Such situations are distinct from instances of coercion where women hide their decisions and contraceptive use from their partners to evade sanctions. These motivations should be considered in future research on covert use, as they are likely to inform different contraceptive dynamics and be associated with different consequences in case of disclosure.

In addition to the issue of autonomy, covert use presents another dilemma in the conceptualization of women’s empowerment, as women who are using contraception covertly may be unable to voice their preferences, but are ultimately able to exercise their choices, thereby achieving their reproductive goals. From an individual perspective, women act according to their preferences, while from a couple perspective, women and men are unable to negotiate and find agreement on their reproductive preferences. Family planning programs that engage male partners may improve couple communication and increase agreement on reproductive decisions, but should also protect and support women’s ability to prevent pregnancy, regardless of partner engagement.

While covert use represents some form of agency over contraceptive practices, it also presents with a number of challenges women must overcome to continue using a method. Managing side effects, including the financial burden of seeking care from a health provider, was a particular challenge in the absence of partner financial and social support [9]. Universal healthcare coverage, including the free provision of contraception and management of contraceptive-related side effects, could help women overcome some of these barriers. More generally, women’s covert contraceptive experience was often laden with concerns of being discovered or suspected by their partners [28, 46]. Justifying a non-pregnant status, while in a sexually active union often came with a socially undesirable label of being infertile**,** as seen in other pronatalist societies [47]. In addition, the price of being discovered was high, as many women were perceived as transgressing the social order. Considered unfaithful or untrustworthy in case of discovery, many women feared partnership dissolution and the loss of financial support if discovered using contraception covertly. With such looming fears of consequences of discovery, covert use also limits method choice for women. While some women may prefer other options, their range is limited to those methods that have clandestine use abilities. Previous research has shown that women in relationships where intimate partner violence prevails are unlikely to use and continue use of their preferred methods [48, 49].

The study is not without limitations. While we sought to explore women’s decisions to use contraception covertly across a variety of economic and cultural settings in sub-Saharan Africa, our results are not transferrable to the entire populations within those countries because of intra- and inter-country cultural variations. In addition, our exploration of reasons and consequences of using contraception covertly may not have captured the complexity of women’s experiences, given that covert use of family planning was not the primary focus of the broader WGE-SRH study. Therefore, information about specific strategies for concealing contraceptive use and their implications for consistent and continuous use, or the ways in which women disclose use, may not have been fully considered within the present study. Translating the audio files to English during the transcription may affected data quality, however, research assistants in each site were carefully recruited based on local and English language proficiency to minimize this limitation. Further, a quality control process to ascertain proper translation and transcription of the audio recordings by independent persons was done on a set of audio files.

This study provides a cross-cultural perspective that can improve existing programs and policies related to family planning. Specifically, the results of this study underscore the need for sexual educational programs that target men and boys to respect and support women’s reproductive decisions. At the macro-level, universal coverage of family planning is central to minimizing the economic burdens many women encounter when they lack partner support. This study provides a more nuanced perspective on the meaning of covert use, as both a sign of constraint and a marker of women’s reproductive empowerment. Future quantitative research is encouraged to examine the role of covert use in contraceptive dynamics, including method switching and discontinuation; continued qualitative research is needed to understand men’s myths and misperceptions of family planning that may increase couple disagreement, as well as further exploration of traditional methods, namely fertility awareness methods, as covert options.

## Conclusions

Our study contributes a deeper understanding of the dynamics of covert contraceptive use, indicating that the practice is common across diverse social contexts in sub-Saharan Africa. Covert use is laden with several challenges that impede method continuation, including lack of resources and social support, especially when women experience side effects related to their contraceptive use. Women’s fear of physical, social, and financial consequences of their covert use being discovered further constrains women autonomy in reproductive decisions. However, in several instances, covert use also indicates women taking independent action without involving men, and therefore, should not always be seen as an indicator of limited empowerment or even disempowerment. Programs aimed at sustaining voluntary contraceptive use will be more effective if they fully understand the dynamics of covert contraceptive use and establish context-specific strategies to support women, taking into account their motivations and challenges surrounding covert use of contraception.

## Data Availability

The datasets generated and/or analyzed during the current study are not publicly available due to the qualitative nature of the data and identifying geographic information, but are available from the corresponding author on reasonable request.

## Abbreviations

DHS: Demographic and Health Surveys
FGD: Focus group discussion
IRB: Institutional Review Board
IDI: In-depth interview
JHSPH: Johns Hopkins Bloomberg School of Public Health
LMIC: Low- and middle-income country
PMA2020: Performance Monitoring and Accountability 2020
SRH: Sexual and reproductive health
WGE: Women’s and girls’ empowerment
WGE-SRH: Women’s and Girls’ Empowerment in Sexual and Reproductive Health Study
